# Immunohistochemical and Ultrastructural Characterization of Telocytes in Normal and Diabetic Human Kidneys

**DOI:** 10.3390/biom14080968

**Published:** 2024-08-08

**Authors:** Sabrina Valente, Marta Villacampa Lahoz, Francesco Vasuri, Gianandrea Pasquinelli

**Affiliations:** 1 Department of Medical and Surgical Sciences (DIMEC), University of Bologna, 40138 Bologna, Italy; martalahoz99@gmail.com (M.V.L.); francesco.vasuri@gmail.com (F.V.); gianandr.pasquinelli@unibo.it (G.P.); 2 IRCCS Azienda Ospedaliero-Universitaria di Bologna, 40138 Bologna, Italy

**Keywords:** telocyte, human kidney, immunohistochemistry, transmission electron microscopy, diabetic nephropathy

## Abstract

**Background**: Telocytes are interstitial stromal cells identified in various human organs, including the kidney. Their presence and role in human diabetic kidney disease remain unknown. **Methods**: To identify and localize telocytes in glomerular and tubule-interstitial compartments, both normal and diabetic human renal tissues were examined using immunohistochemistry, immunofluorescence, and transmission electron microscopy. **Results**: Renal telocytes are elongated interstitial cells with long, thin telopodes, showing alternating thin and thick segments. They expressed CD34, Nestin, α-SMA, and Vimentin markers. Occasionally, c-Kit expression was observed in some rounded and spindle cells, while no positivity was detected for PDGFR-β and NG2. Telocytes were identified around Bowman’s capsule, tubules, and peritubular capillaries in both normal and diabetic conditions. In diabetic renal samples, there was a significant increase in α-SMA expressing telocytes, leading to periglomerular fibrosis. These telocytes also exhibited a synthetic phenotype with proteoglycan deposition in the extracellular matrix and, in some cases, showed pre-adipocytic differentiation. **Conclusions**: Telocytes were identified in normal and diabetic human kidneys. These cells form an elastic mechanical scaffold in the interstitium and are present in all renal cortical compartments. In diabetic samples, their increased α-SMA expression and synthetic phenotype suggest their potential role in the pathogenesis of diabetic nephropathy.

## 1. Introduction

Classically, histological studies of the kidney cortex have highlighted the existence of cellular networks formed by two main components: interstitial fibroblasts and dendritic reticulum cells [1]. However, using additional immunohistochemical markers such as CD34, c-Kit, Nestin, α-smooth muscle actin (α-SMA), and Vimentin, the presence of a third network in the renal cortical interstitium can be identified. This network is constituted of telocytes.

Telocytes (TCs) are a distinct population of stromal/interstitial cells first described by the research groups of Popescu and Faussone-Pellegrini in 2010, primarily using transmission electron microscopy (TEM), which is considered the gold standard for identifying TCs [2,3]. TCs exhibit a characteristic morphology that distinguishes them from other cell types: they have a small cell body with an oval nucleus, sparse cytoplasm, and very long, thin, and beaded cytoplasmic processes called telopodes. These telopodes consist of extremely thin segments (podomeres) alternating with thicker portions (podoms), which contain mitochondria, endoplasmic reticulum, and caveolae [4]. TCs form intricate networks, establishing contact with various other cells, vessels, and nerves [5].

Over the years, TCs have been identified in a wide range of human organs and tissues, including the heart [6,7], gastrointestinal tract [8,9], blood vessels [10,11], female and male reproductive systems [12,13,14,15], skin [16,17], glands [18,19,20], and kidney [21,22]. These findings have been primarily disclosed using TEM, scanning electron microscopy (SEM), immunofluorescence, and immunohistochemistry.

Given the absence of a unique marker for TCs [23], these cells are identified by different molecules depending on the tissue in which they are found. Using immunohistochemical approaches, TCs are commonly recognized through markers such as CD34, c-Kit, Vimentin, platelet-derived growth factor receptor (PDGFR)-α, and α-SMA, which are variably expressed across different organs and even within the same organ and tissue [4,9,23,24]. Additionally, TCs have been reported to express Nestin, PDGFR-β, and VEGF [15,22,25]. The phenotype plasticity is more complex and trickier in the oncological contest [26]. The immunophenotypic characterization of TCs is often combined with double immunofluorescence to facilitate their identification.

Several functions have been attributed to TCs, though many of these roles remain to be fully clarified. TCs are believed to play a pivotal role in maintaining organ structure and mechanical sensing [4], facilitating cell-to-cell communication and intercellular signaling through homocellular or heterocellular connections with neighboring cells [5,23,27], and mediating paracrine effects via the release of exosomes and extracellular vesicles [28,29]. They are also implicated in immune response regulation [30,31], angiogenesis [32], reparative and regenerative processes [33,34,35], and stem cell functions [36]. Additionally, evidence suggests that TCs may be involved in various pathological processes, including tumors, aortic aneurysms, fibrosis, chronic inflammation, and skin and heart diseases [26,37,38,39,40,41,42]. All these conditions are chronic diseases sharing the remodeling of the extracellular matrix components whose cell determinants are not completely understood. Diabetes belongs to this spectrum of diseases; it is a chronic disease with significant remodeling of the extracellular matrix of the basal lamina.

To our knowledge, the presence of TCs in human diabetic nephropathy has not been previously described. Diabetic nephropathy is characterized by morpho-functional changes such as glomerular basement membrane thickening, mesangial expansion, podocyte alterations, tubular damage, a decline in kidney function, renal impairment and, eventually, end-stage renal disease. Therefore, the potential involvement of TCs in diabetic nephropathy remains unclear.

The aim of this study is to investigate the presence and localization of TCs in normal human kidney tissue and provide the first evidence of their presence in diabetic kidneys using light and transmission electron microscopy (TEM) techniques. Furthermore, this study seeks to define the potential role of TCs in diabetic nephropathy, particularly considering the disease’s progression towards fibrosis, chronicity, and organ failure.

## 2. Materials and Methods

This retrospective study was performed on archival paraffin sections of normal (*n* = 2) and diabetic (*n* = 7) human renal tissues and on resin ultrathin sections and conducted in accordance with the Declaration of Helsinki, and approved by Local Ethics Committee (protocol number RecoverEMO; 185/2020/Sper/AOUBo).

### 2.1. Light Microscopy

To investigate the immunophenotypic profile of TCs, immunohistochemistry and double immunofluorescence techniques were employed. For immunohistochemistry, the NovoLink™ Polymer Detection System (Leica Biosystems, Newcastle Upon Tyne, UK) was used according to the manufacturer’s datasheet. Three-μm-thick sections of formalin-fixed or Serra’s solution, paraffin-embedded tissues were deparaffined in xylene, rehydrated with ethanol at decreased concentrations, and washed in distilled water. To unmask antigens, samples were treated with heat by autoclaving in a citrate buffer pH = 6 at 120 °C for 20 min, followed by cooling for 20 min and washing in distilled water.

To neutralize the endogenous peroxidase activity, sections were exposed to 3% H_2_O_2_ (Sigma-Aldrich, St Louis, MO, USA) in absolute methanol (Sigma-Aldrich) for 5 min at room temperature (RT) in the dark. After washing in Tris Buffered Saline (TBS), the sections were blocked to reduce the nonspecific binding before incubation with CD34 (1:80, class II, clone QBEnd-10, Dako, Glostrup, Denmark), c-Kit (1:200, clone H-300, Santa Cruz Biotechnology, Dallas, TX, United States), α-SMA (1:9000, clone 1A4, Sigma-Aldrich), Nestin (1:400, clone 10c2, Santa Cruz Biotechnology), Vimentin (1:500, clone V9, Santa Cruz Biotechnology), PDGFR-β (1:200, clone PR7212, R&D Systems, Minneapolis, MN, USA), and Neuron glial antigen 2 (NG2) (1:50, clone LHM-2, R&D Systems) primary antibodies diluted in 1% bovine serum albumin (BSA) in PBS overnight at 4 °C. Negative control was performed by omitting the primary antibodies. Then, sections were treated with 3,3′-diaminobenzidine (DAB) substrate/chromogen, counterstained with hematoxylin, dehydrated in ethanol, and passed in xylene before mounting the coverslip. All incubations were performed in a wet chamber. Samples were observed with a Leitz Diaplan light microscope (Wetzlar, Germany), acquiring digital imaging using Image-Pro Plus 6 software (Media Cybernetics, Rockville, MD, USA). The semiquantitative analysis was performed by two expert pathologists; it was based on the positivity or negativity and intensity of immunostaining for the CD34, Nestin, α-SMA, Vimentin, and c-Kit of normal and diabetic human renal samples. A score was attributed to each renal compartment (glomerulus and tubule-interstitium) as well as to each renal component (Bowman’s capsule, mesangial cells, glomerular endothelial cells, peritubular capillaries, tubules, and interstitium). To quantify CD34- and α-SMA-positive areas, ten random images were acquired from each sample at 40x of magnification and measured using Image-Pro Plus 6 measurement tools. Values were expressed as the mean of the positive area/total area ratio ± standard deviation (SD) and relative percentages.

For double immunofluorescence, additional sections of both normal and diabetic human renal tissues were used for detecting CD34 and c-Kit expression. After dewaxing and rehydration, tissue sections were blocked with 1% PBS/BSA for 30 min at RT and subsequently labeled with a solution of CD34 (1:80, Dako) and c-Kit (1:200, Santa Cruz Biothecnology) primary antibodies in 1% PBS/BSA for 1 h at 37 °C. After several rounds of washing with PBS, tissues were stained with a solution of Alexa Fluor 488 (1:250, ThermoFisher Scientific, Carlsbad, CA, USA) and Alexa Fluor 546 (1:250, ThermoFisher Scientific) secondary antibodies in 1% PBS/BSA for 1 h at 37 °C in the dark, and washed and counterstained with the ProLong™ Gold Antifade reagent with DAPI (ThermoFisher Scientific); all incubations were performed in a wet chamber. Sample examinations and digital image acquisitions were performed using a Leica DMI4000 B-inverted fluorescence microscope (Leica Microsystems, Wetzlar, Germany).

### 2.2. Transmission Electron Microscopy

Ultrathin sections were obtained during the routine examination. Briefly, renal tissues were fixed in cacodylate, buffered 2.5% glutaraldehyde overnight at 4 °C, post-fixed in osmium tetroxide, dehydrated with ethanol, and embedded in Araldite resin (Serva, Heidelberg, Germany). Resin blocks were cut with an ultramicrotome (Ultracut, Reichert, Vienna, Austria) to obtain ultrathin sections and, after counterstaining with uranyl acetate followed by lead citrate, they were viewed in a Philips CM100 (FEI Company, ThermoFisher, Waltham, MA, USA) Transmission Electron Microscope equipped with a digital camera for image acquisitions.

### 2.3. Statistical Analysis

Statistical analysis was performed using GraphPad Prism 8 software. Statistical differences between normal and diabetic human renal tissue samples were evaluated using unpaired Student’s *t*-test Values are expressed as means ± standard deviations (SD); *p* values < 0.05 were considered statistically significant.

## 3. Results

### 3.1. Immunophenotype and Localization of Telocytes in Normal and Diabetic Human Kidney Tissues

To identify TCs in renal tissues, an immunohistochemical analysis was performed using markers such as CD34, Nestin, α-SMA, Vimentin, and c-Kit. This analysis revealed the presence of spindle-shaped cells with long, thin prolongations—characteristic of TCs—localized in periglomerular and pericapillary glomerular areas as well as in the interstitium of both normal and diabetic human renal tissues. These findings are summarized in Table 1.

**Figure 1:**
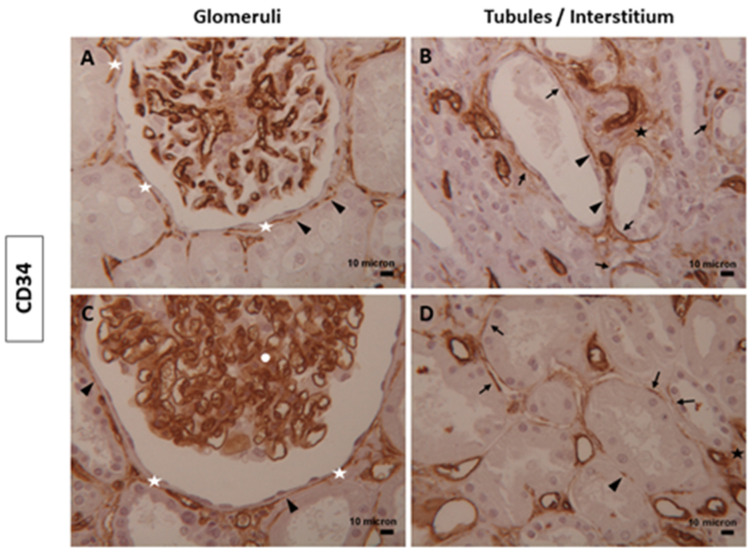
TCs expressing CD34 in human renal tissues. Representative images of TCs positive for CD34 localized in glomeruli and in tubules/interstitium compartments of (**A**,**B**) normal and (**C**,**D**) diabetic human kidney tissues. TCs and their telopodes immunostaining for the CD34 marker were localized along the renal Bowman’s capsule (white asterisks), in mesangial cells (white circle), around some tubules (dark arrows), in endothelial cells of glomerular and peritubular capillaries and in the interstitium (black asterisks). TCs with dot positivity (black arrowheads) were also found. Magnification of images = 25x. Scale bars: 10 µm.

Renal TCs expressing CD34 were detected along Bowman’s capsules (Figure 1A,C), around some tubules, and in the interstitium (Figure 1B,D); as expected, CD34 intensely stained endothelial cells of glomerular and peritubular capillaries (Figure 1) in both conditions. The glomerular mesangial cells positive for CD34 were seen only in diabetic samples (Figure 1C). Furthermore, we noted the presence of numerous intensely stained TCs for CD34 forming multilayers around the Bowman’s capsule in some diabetic tissues.

**Figure 2:**
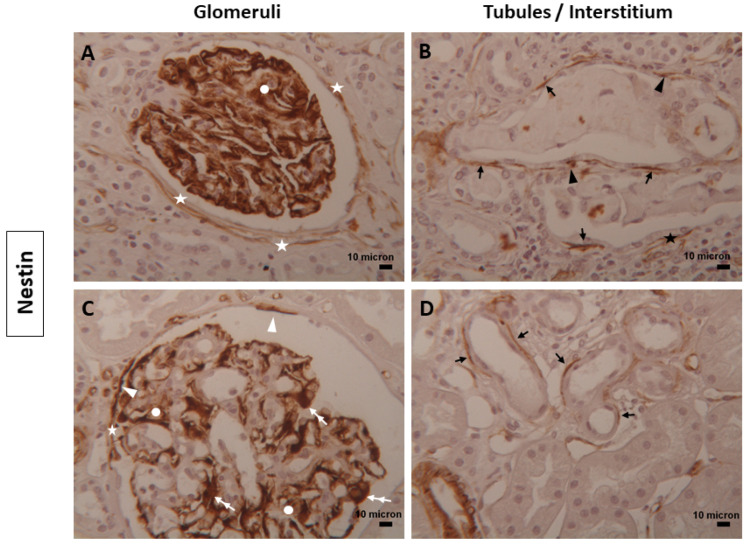
TCs expressing Nestin in human renal tissues. Representative images of TCs and their projections positive for Nestin were detected along Bowman’s capsule (white asterisks), around renal tubules (dark arrows), and in the interstitium (black asterisks) of (**A**,**B**) normal and (**C**,**D**) diabetic human kidney tissues. Dot positivity (dark arrowheads), as well as parietal epithelial cells (white arrowheads), podocytes (double white arrows), and mesangial cells (white circles) positive for Nestin were also seen. Magnification of images = 25x. Scale bars = 10 µm.

Nestin staining was diffuse and strongly positive in podocytes, mesangial cells, and the endothelial cells of glomerular capillaries in normal renal tissues (Figure 2A,C). In diabetic renal tissues, intense Nestin staining was limited to parietal epithelial cells, podocytes, a few mesangial cells, and glomerular endothelial cells (Figure 2A,C). The reduction in mesangial Nestin staining in diabetic samples was attributed to increased matrix production. In normal kidneys, Bowman’s capsule was lined with long portions of renal TCs expressing Nestin (Figure 2A). In contrast, diabetic tissue showed only partial coverage by fragmented telopodes (Figure 2C). In the tubulo-interstitial compartment, Nestin-positive TCs surrounded some tubules in both normal and diabetic conditions (Figure 2B,D).

**Figure 3:**
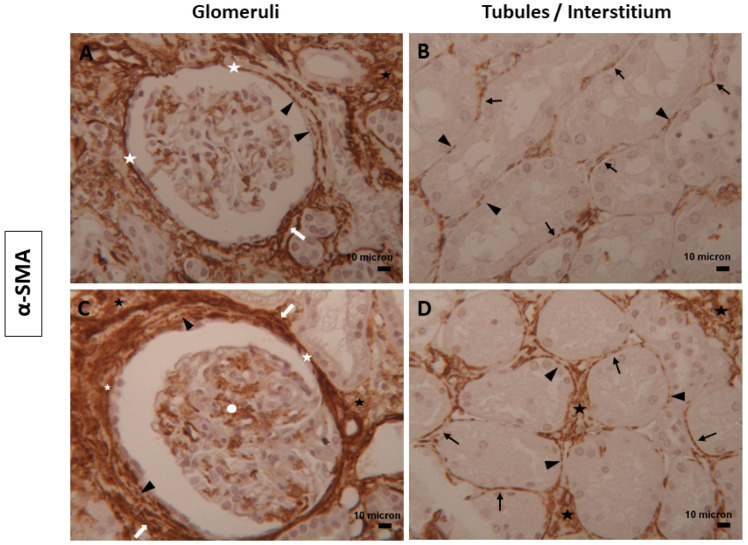
TCs expressing α-SMA in human renal tissues. Representative images of TCs positive for α-SMA distributed in the glomeruli and tubules/interstitium compartment of (**A**,**B**) normal and (**C**,**D**) diabetic human kidney tissues. TCs with their long and thin projections were localized along Bowman’s capsule (white asterisks) in renal tubules (dark arrows), interstitium (black asterisks), and mesangial cells (white circle). The dot positivity of TCs (dark arrowheads) and their organization as multilayers (white arrows) were also seen. Magnification of images = 25x Scale bars = 10 µm.

The immunoreaction for α-SMA, a contractile protein, was observed in all renal compartments examined (Figure 3). In normal kidneys, α-SMA positivity was present in some mesangial cells (Figure 3A), whereas in diabetic tissues, the expression of the α-SMA marker was more intense and diffusely distributed (Figure 3C). In both renal conditions, TCs intensely stained for α-SMA were visible in the outer region of Bowman’s capsule, which was entirely surrounded by these cells and their long, thin cytoplasmic prolongations (Figure 3A,C). Numerous TCs were arranged in a multilayer pattern in this location, with a more pronounced presence in diabetic samples (periglomerular fibrosis) (Figure 3C). Several renal tubules were lined by α-SMA-positive TCs, which appeared thicker in diabetic samples compared to normal ones (Figure 3B,D). In the interstitium, α-SMA-positive TCs were organized into a widespread and intricate network and were also located around peritubular capillaries (Figure 3).

**Figure 4:**
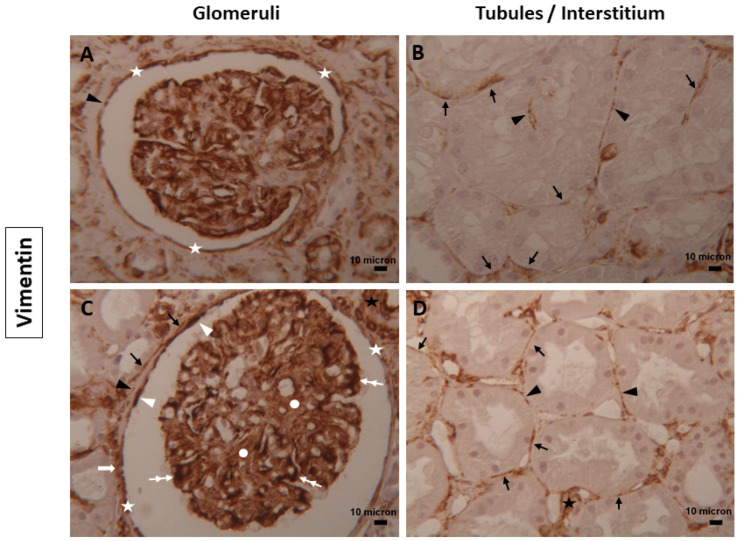
TCs expressing Vimentin in human renal tissues. Representative images of TCs and their telopodes positive for Vimentin in the glomeruli and tubules/interstitium compartment of (**A**,**B**) normal and (**C**,**D**) diabetic human kidney tissues. They were detected in the entire outer boundary of Bowman’s capsule (white asterisks), around some tubules (dark arrows), and in the renal interstitium (black asterisks). The dot positivity (dark arrowheads), small multilayers (white arrows), parietal epithelial cells (white arrowheads), podocytes (double white arrows), and mesangial cells (white circles) positive for Vimentin were also seen. Magnification of images = 25x. Scale bars = 10 µm.

Renal TCs expressing Vimentin were identified along the entire outer boundary of Bowman’s capsule and in endothelial cells of glomerular capillaries (Figure 4A,C), as well as around some tubules whose expression increased in number and intensity in diabetic samples, in peritubular vessels and in the interstitium (Figure 4B,D) of both groups; parietal epithelial cells, podocytes and mesangial cells positive for Vimentin-were more marked in diabetic (Figure 4C) than normal samples (Figure 4A). Some multilayers of TCs expressing Vimentin were also detected.

**Figure 5:**
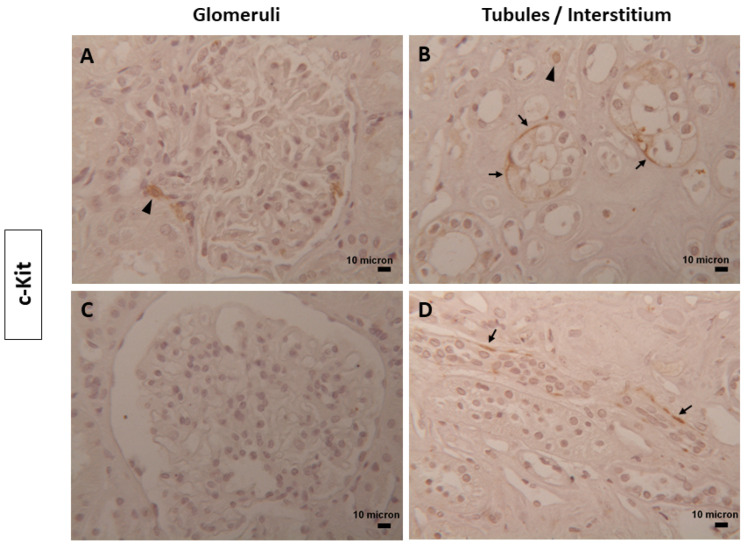
c-Kit in human renal tissues. Representative images of occasional cells positive for c-Kit in the glomeruli and tubules/interstitium compartment of (**A**,**B**) normal and (**C**,**D**) diabetic human kidney tissues. A few spindle cells, ascribable to TCs, were localized around some tubules (black arrows); at the same sites, rare, rounded cells were also seen (black arrowheads). Magnification of images = 25x. Scale bars = 10 µm.

Occasional cells expressing the c-Kit marker were identified in both normal and diabetic conditions (Figure 5). These cells exhibited a dual morphology: some appeared rounded (Figure 5A,B), indicative of mast cells, an inflammatory cell type observed in both glomeruli and the interstitium, while elongated cells, characteristic of TCs, were detected along a few tubules (Figure 5B–D).

Immunolabeling for PDGFR-β and NG2 markers was negative in both renal tissues. Therefore, no pericytes were observed immunohistochemically around the interstitial peritubular capillaries.

In the renal tissues examined, TCs immunostained for CD34, Nestin, α-SMA, and Vimentin showed double positivity: linear and dot. The dotted positivity could reflect the telopodes structure composed of alternating thin (podomeres) and dilated (podoms) segments that, when too thin, were not observable under the light microscope (Figure 1, Figure 2, Figure 3 and Figure 4).

**Figure 6:**
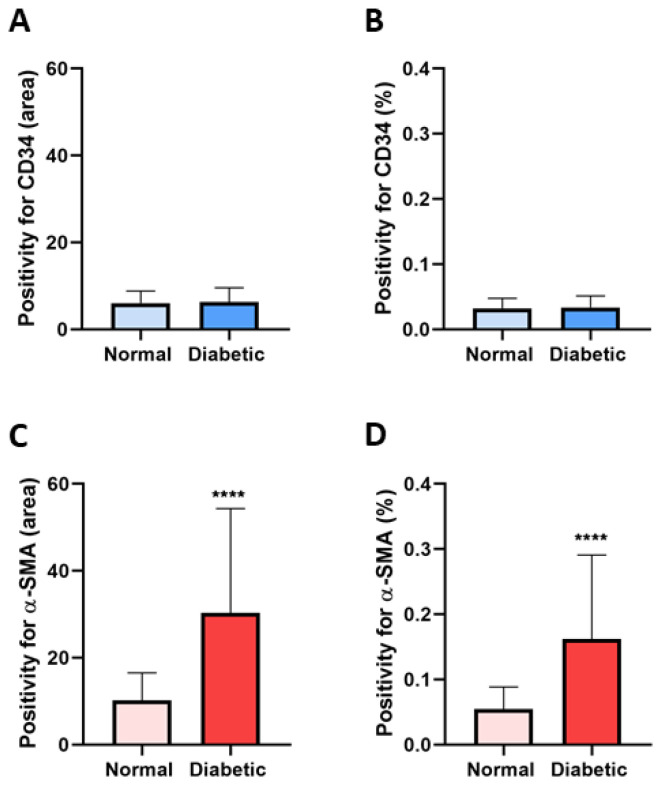
Quantitative analysis of CD34- and α-SMA-positive expression. The quantification of the (**A**) area or (**B**) percentage occupied by TCs positive for CD34 was almost superimposable in both normal and diabetic renal tissues without significant differences. (**C**) The area or (**D**) percentage of TCs expressing α-SMA was significantly increased in diabetic tissues compared to normal samples. Values are expressed as mean ± SD. **** *p* value < 0.0001.

The quantitative analysis for CD34 positivity showed a slight increase in diabetic tissues (mean positive area: 6.266 ± 3.306; mean percentage of positivity: 0.03354 ± 0.01769) than normal samples (mean positive area: 6.041 ± 2.805; mean percentage of positivity: 0.03233 ± 0.01501) without significant differences (Figure 6A,B). Of interest was α-SMA, whose expression significantly increased in diabetic tissues (mean positive area: 30.33 ± 24.02; mean percentage of positivity: 0.1623 ± 0.1286) in contrast to normal ones (mean positive area: 10.19 ± 6.358; mean percentage of positivity: 0.05452 ± 0.03403) (Figure 6C,D).

**Figure 7:**
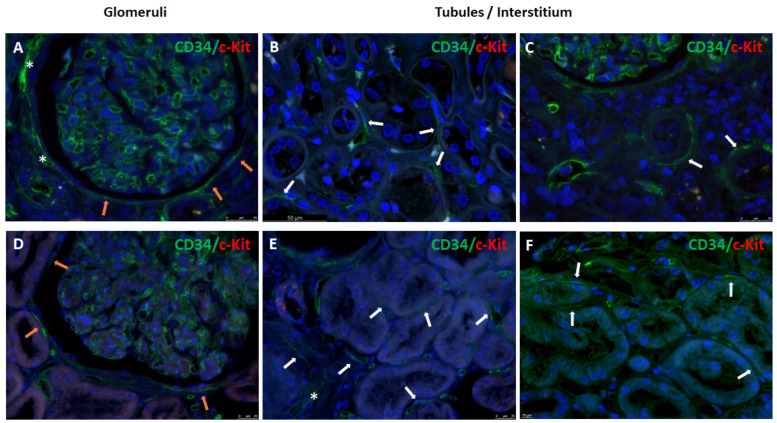
Double immunofluorescence for CD34 and c-Kit markers in human renal tissues. Representative images of TCs positive for CD34 distributed in glomeruli and in the tubules/interstitium compartment of (**A**–**C**) normal and (**D**–**F**) diabetic human kidney tissues. CD34-stained TCs and their elongated and slim projections were localized along Bowman’s capsule (orange arrows) and in some tubules (white arrows), showing linear and dot positivity; multilayers of TCs expressing CD34 (white asterisks) were also seen in some cases. (**A**,**C**–**E**) scale bars = 25 µm; (**B**) scale bar = 50 µm; and (**F**) scale bar = 10 µm.

Double immunofluorescence for CD34 and c-Kit markers was additionally performed. The immunostaining confirmed the presence of renal TCs positive for CD34 and characterized by oval nuclei and long and thin cytoplasmic extensions. TCs were localized around Bowman’s capsule, tubules, peritubular capillaries, and in the interstitium of normal (Figure 7A–C) and diabetic human renal kidneys (Figure 7D–F); endothelial cells were also stained; and rare rounded c-Kit positive cells were exclusively detected in a few renal tubules.

### 3.2. Ultrastructure of Telocytes in Normal and Diabetic Human Kidney Tissues

**Figure 8:**
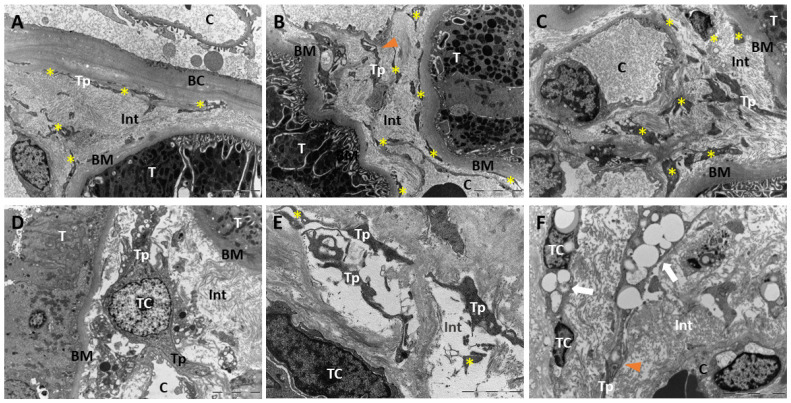
Ultrastructural identification and localization of TCs in normal and diabetic human renal tissues. (**A**–**C**) Ultrastructure of TCs in normal kidney tissues. Several long and slender telopodes and their fragments were identified along the outer side of Bowman’s capsule, near the basal membrane of tubules and close to peritubular capillaries (Tp and yellow asterisks); in interstitium were visible thin segments (podomeres) that alternated to thick (podoms) regions (orange arrowheads). (**D**–**F**) Ultrastructure of TCs in diabetic kidney tissues. TCs with spindle nuclei and long and thin telopodes were identified along the basal membrane of tubules near peritubular capillaries in the interstitial space. Focally, TCs were embedded in extracellular lacunae containing proteoglycan particles (**E**); in a few cases, telopodes were packed with lipid droplets ((**F**), white arrows). Abbreviations: BC: Bowman’s capsule; BM: basal membrane of tubules. C: capillary; Int: interstitium; T: tubule; TC: telocyte; and Tp: telopodes. (**A**–**D**,**F**) scale bars = 5 µm. (**E**) Scale bar = 2 µm.

TEM investigation revealed the presence of spindle cells with cytoplasm long, slender prolongations features typical of TCs in both normal and diabetic human kidney tissues (Figure 8). Very long and extremely thin fragmented telopodes were located along the outer side of Bowman’s capsule (Figure 8A) and in the interstitium along the basal membrane of tubules (Figure 8B) and near the peritubular capillaries (Figure 8C), which occasionally displayed a moniliform appearance due to the alternation of podomeres and podoms (Figure 8B,C). In diabetic renal tissue, the cytoplasm of the TCs exhibited an expansion of the rough endoplasmic reticulum (Figure 8D), and at high magnification, it could be observed that the TCs were frequently embedded in electron-transparent extracellular lacunae where proteoglycan particles were present (Figure 8E); occasionally, the cytoplasm of the TCs contained single or multiple lipid droplets (Figure 8F).

## 4. Discussion

This study provides evidence of the presence of TCs in the human renal cortex of both normal and diabetic samples by combining immunohistochemical and double immunofluorescence staining with TEM techniques. Alongside fibroblasts and potential dendritic reticulum cells [1], we observed cells with typical morphology and immunophenotypical features of TCs, including very long and slender cytoplasmic telopodes with alternating podomeres and dilated podoms. The previous documentation of TCs in the human kidney focused solely on normal renal samples [21,22]. Here, we provide the first evidence of TCs, their localization, and distribution in diabetic human kidney tissues.

Among the morphological methods, TEM is considered the gold standard for distinguishing TCs from other interstitial/stromal cells [3]. Ultrastructural analysis highlighted TCs and fragments of their long, thin telopodes in both normal and diabetic human kidneys. TCs were primarily located around Bowman’s capsule and in the interstitium, along tubule basal membranes, and close to peritubular capillaries. Podoms containing cytoplasmic organelles, such as mitochondria, were also observed. This organizational pattern and the morphological features of TCs can be exclusively observed via TEM analysis. Our observations suggest that TCs form a geometric scaffold in the renal cortex and likely in other tissues where they have been described, ensuring the mechanical stability of the interstitium while providing a lightweight, highly stable structure resistant to internal and external mechanical stress.

Over the years, various markers have been tested without identifying one that is specific for TCs. Therefore, their characterization requires a combined study integrating TEM with immunohistochemistry. Surface and intracellular molecules, including CD34, c-Kit, Vimentin, PDGFR-α, and α-SMA, have been proposed to identify TCs [23]. The expression of these markers varies depending on the anatomical site and even within the same organs and tissues [23,24,25], reflecting the phenotypic plasticity of TCs, which is a trait common to other stromal cells, such as mesenchymal stromal cells.

In this study, renal TCs were positive for CD34, α-SMA, Vimentin, and Nestin, with variable staining intensity. The immunohistochemical panel identified TCs in the periglomerular area, along the outside of Bowman’s capsule, and in the interstitial area around tubules and interstitial peritubular capillaries in both normal and diabetic renal tissues. These results were consistent with TEM findings. For c-Kit, a member of the receptor tyrosine kinase family involved in several biological processes, our results show the presence of rare spindle and rounded cells in Bowman’s capsule membrane and some tubules, partially confirming previous reports [21,22]. The low or absent expression of c-Kit was further confirmed through double immunofluorescence staining for CD34/c-Kit.

Given the presence of TCs near peritubular capillaries, it was necessary to distinguish TCs from classical mural perivascular cells, such as pericytes. The immunohistochemical analysis minimized the presence of pericytes in the stromal cortical tissue architecture due to the absence of PDGFR-β and NG2, which are two classical pericyte lineage markers, in the peritubular capillaries of the interstitium.

Notably, immunolabeled TCs displayed a dual morphology: linear and/or dot. This aspect reflects the internal organization of telopodes, as evidenced by ultrastructural studies, showing a moniliform shape due to the alternation of podomeres (thin regions) and podoms (thick portions) consistent with previous studies on in vitro human TCs culture [6,31]. When these extensions become extremely thin, they may not be detectable under a light microscope, unlike the dilated portions, which are observable. This morphology supports the tensional structure function of TCs, providing cellular flexibility adaptable to changes in stromal rigidity.

Additionally, we observed multiple TCs arranged in concentric layers, with intensely immunostained multilayers being more pronounced in diabetic human kidney tissues than in normal ones. This feature corresponds to periglomerular fibrosis, which is known to correlate with interstitial fibrosis and a decline in renal function [43].

Quantifying the immunostaining of TCs for CD34 and α-SMA revealed a significant increase in α-SMA positivity in diabetic tissues compared to normal kidneys, suggesting a potential role of TCs in renal pathologies. Previous studies have shown that α-SMA expression in the glomeruli and interstitial areas of human biopsies affected by various kidney diseases is associated with reduced renal function and disease progression, indicating that α-SMA expression could be a useful diagnostic and/or prognostic factor [44,45,46,47,48,49].

TCs have been attributed several functions, including structural support [4], communication [5,23,27,28,29], immunomodulation [30,31], angiogenesis [32], reparative and regenerative processes [33,34,35], and the regulation of stem/progenitor cells in stem cell niches of various organs [36]. In damaged human kidneys, stromal cells acquire a myofibroblast phenotype characterized by α-SMA expression and collagen and matrix production [1]. The increased α-SMA expression in diabetic conditions raises questions about chronicity and the derivation of myofibroblasts in the pathological renal interstitium. While the direct transformation of fibroblasts into myofibroblasts remains unproven, the potential derivation of myofibroblasts from TCs cannot be ruled out due to the noted plasticity of TCs.

In the TEM analysis of diabetic kidney samples, we observed several morphological variations in TCs, including an increase in synthetic components, such as expanded rough endoplasmic reticulum and the formation of electron-transparent niches containing proteoglycans. These aspects indicate that in a pathological context like diabetes, TCs may assume synthetic capabilities and influence the extracellular matrix composition. The presence of proteoglycans, particularly versican, in other pathological contexts precedes collagen accumulation. Notably, in patients with cardiomyopathy, versican protein levels extended from the perivascular region into the tissue interstitium [50]. Although we did not study versican’s presence in the renal cortical tissues of normal and diabetic subjects, this hypothesis warrants further investigation as it could constitute a therapeutic target to prevent fibrosis onset.

Further evidence of TCs’ plasticity was provided by the ultrastructural documentation of cytoplasmic lipid droplets in interstitial TCs in our diabetic samples. Based on this observation, we speculate a potential pre-adipocyte differentiation of cellular portion, possibly resulting from either the direct differentiation of TCs or induced fibroblasts, so-called lipo-fibroblasts, or other mesenchymal stromal cells differentiating into adipogenic lineage under certain conditions. Further investigations are necessary to better elucidate the plasticity and role of TCs in diabetic nephropathy.

## 5. Conclusions

In summary, our study documents that TCs positive for CD34, α-SMA, Nestin, and Vimentin markers are present in both normal and diabetic human kidney tissues using combined immunohistochemical, immunofluorescence, and ultrastructural investigations. Due to their unique morphological characteristics, TEM remains the gold standard technique for their characterization, allowing an appreciation of the structural organization of telopodes. TCs were distributed in the interstitium along Bowman’s capsule, around tubules, and peritubular capillaries, forming an elastic mechanical scaffold. In diabetic samples, multilayers of TCs constituted periglomerular fibrosis, which was a feature frequently observed. Their plasticity, with increased α-SMA expression and the acquisition of a synthetic phenotype or pre-adipocytic commitment, suggests their potential role in the pathogenesis of diabetic nephropathy. Further studies are necessary to elucidate the precise role of TCs in diabetic nephropathy and to develop new therapies for managing this pathological condition.

## Figures and Tables

**Table 1:** Immunophenotypic profile of TCs and their localization in human kidney cortical tissues.

	Glomerulus						Tubule-Interstitium					
	Bowman’s Capsule		Mesangial Cells		Glomerular Endothelial Cells		Peritubular Capillaries		Tubules		Interstitium	
**Antibodies**	N	D	N	D	N	D	N	D	N	D	N	D
**CD34**	+	+	-	+	++	++	++	++	+	+	+	+
**Nestin**	+	+	++	+	++	+	+/-	+	+	+	+/-	+/-
**α-SMA**	++	++	+	+	+/-	+/-	+	++	+	++	++	++
**Vimentin**	++	++	+	+	++	++	+	++	+	++	+	++
**c-Kit**	+/-	-	-	-	-	-	-	-	+/-	+/-	-	-

Abbreviations: N = normal human kidney; D = diabetic human kidney. ++ = strong and diffuse positivity; + = strong and focal positivity; +/- = occasional positivity; and - = negative staining.

## Data Availability

All relevant data are available within the manuscript.
